# Morphological and Hemodynamic Risk Factors for Middle Cerebral Artery Aneurysm: a Case-Control Study of 190 Patients

**DOI:** 10.1038/s41598-019-56061-2

**Published:** 2020-02-06

**Authors:** Wojciech Kaspera, Karolina Ćmiel-Smorzyk, Wojciech Wolański, Edyta Kawlewska, Anna Hebda, Marek Gzik, Piotr Ładziński

**Affiliations:** 1 Department of Neurosurgery, Medical University of Silesia, Regional Hospital, Sosnowiec, Poland; 2 Department of Biomechatronics, Silesian University of Technology, Zabrze, Poland; 3 Maria Skłodowska-Curie Memorial Cancer Center and Institute of Oncology, Gliwice, Poland

**Keywords:** Pathology, Brain imaging

## Abstract

This study analyzed morphometric and hemodynamic parameters of aneurysmal and non-aneurysmal middle cerebral artery (MCA) bifurcations and their relationship with optimal values derived from the principle of minimum work (PMW). The study included 96 patients with MCA aneurysm and 94 controls. Aneurysm patients presented with significantly higher values of the radius and cross-sectional area of the MCA trunk, angle between the post-bifurcation branches (α angle) and volume flow rate (VFR) and had significantly lower values of junction exponent and pulsatility index than the controls. The Φ_1_ and Φ_2_ angles (angles between the MCA trunk axis and the larger and smaller branch, respectively) and α angle in all groups were significantly larger than the optimal PMW-derived angles. The most important independent predictors of MCA aneurysm were junction exponent (odds ratio, OR = 0.42), α angle (OR = 1.07) and VFR (OR = 2.36). Development of cerebral aneurysms might be an independent effect of abnormalities in hemodynamic and morphometric factors. The risk of aneurysm increased proportionally to the deviation of morphometric parameters of the bifurcation from their optimal PMW-derived values. The role of bifurcation angle in aneurysm development needs to be explained in future research as the values of this parameter in both aneurysm patients and non-aneurysmal controls in were scattered considerably around the PMW-derived optimum.

## Introduction

Published data suggest that hemodynamic stress at arterial bifurcation, where kinetic energy of the moving blood is converted to a pressure energy due to a decrease in flow velocity, might be the principal factor responsible for aneurysm formation, enlargement and rupture^1–3^. This evidence originates from clinical observations of patients who developed *de novo* aneurysms in collateral vessels after iatrogenic ligation of the internal carotid artery (ICA)^4^, or due to occlusion of one or more large extracranial cerebral arteries^5^, or presence of anomalies within the circle of Willis^6–8^. Those clinical findings are consistent with the results of experimental studies. According to Hashimoto *et al*.^9,10^, ligation of one of the common carotid arteries resulted in the development of aneurysms in large collateral arteries at the base of the brain in both rat and monkey model.

The evidence presented above suggests that hemodynamic stress (which according to Ferguson^3^ comprises shear stress, pressure and impulse) generated at the bifurcation in response to collision with the blood stream is the principal contributor to aneurysm development. However, it is still unclear which factors determine the magnitude of the hemodynamic stress at arterial bifurcation. The results of studies with glass vessel models^3,11,12^ and computational fluid dynamics (CFD) studies^13–16^ suggest that the magnitude of the hemodynamic stress and its distribution at arterial bifurcation might depend on the blood flow characteristics, as well as on the bifurcation’s geometry, i.e. bifurcation angle and vascular dimensions. However, the latter observation seems to stay in opposition to one of the fundamental laws that govern the structure of vascular networks, the so-called principle of minimum work (PMW) formulated by Murray^17,18^. According to the PMW, vessel dimensions and bifurcation angles follow a mathematical formula, to provide the lowest possible energy expenditures for circulation maintenance and the lowest possible level of hemodynamic stress. The theoretical Murray’s assumptions on the structure of vascular network were confirmed by angiographic studies which demonstrated that the dimensions of various human blood vessels^19,20^, including intracranial arteries^21^, were consistent with the theoretical optimal values imposed by the PMW.

The aim of our study was to verify whether arterial bifurcations in patients with cerebral aneurysm differ in terms of their morphology and selected hemodynamic parameters from the bifurcations in non-aneurysmal controls. Second, we analyzed if the morphometric parameters of the bifurcations in the patients with aneurysm and controls corresponded well with their optimal PMW-derived values. Since the structure of the circle of Willis imposes a certain geometric pattern, bifurcation morphology in the arteries forming the circle, i.e. basilar artery (BA), ICA and anterior communicating artery (ACoA) complex, does not follow the PMW-derived optimum^22^. Therefore, our study involved the middle cerebral artery (MCA) which, unlike other cerebral arteries predilected to aneurysm formation, i.e. ACoA complex, ICA and BA, is not a part of the circle of Willis.

We designed a case-control study with prospective selection of patients with MCA aneurysm and non-aneurysmal controls to determine selected hemodynamic and morphometric parameters of the MCA bifurcation and to analyze their relationship to the PMW-derived optimal values. To the best of our knowledge, this was one of a few case-control studies which, aside from the morphometric parameters, analyzed also the effect of hemodynamic factors and their relationship with the PMW-derived optimal values on the pathogenesis of cerebral aneurysms.

## Materials and Methods

### Patients

The study included 96 consecutive patients (24 men and 72 women) aged 30–75 years (mean 56 ± 10 years) with unruptured MCA aneurysms diagnosed on three-dimensional computed tomography angiography (3D CTA) between June 2013 and June 2017. Control group was comprised of 94 age- and sex-matched patients with no evidence of intracranial pathologies on 3D CTA, among them 34 men and 60 women with mean age of 53 ± 15 years (range 21–75 years). All patients were referred for 3D CTA to exclude MCA aneurysm suspected based on the result of conventional computed tomography (CT), or to establish the etiology of minor symptoms, such as headache or vertigo. The exclusion criteria from the study were: age under 18 years or over 75 years, presence of multiple cerebral aneurysms (other than mirror MCA aneurysms) or other than aneurysm pathologies in the central nervous system that could have a potential effect on cerebral blood flow (e.g. ischemic stroke, intracerebral or subarachnoid hemorrhage), severe systemic disorders (e.g. neoplastic disease), severe heart failure or multi-organ failure, hemodynamically significant stenosis of the extracranial segment of the ICA, pregnancy and family history of cerebral aneurysm or genetically determined conditions associated with increased risk of cerebral aneurysm development, such as autosomal dominant polycystic kidney disease, neurofibromatosis type I, Marfan syndrome, multiple endocrine neoplasia type I, pseudoxanthoma elasticum, hereditary hemorrhagic telangiectasia, and type II and type IV Ehlers-Danlos syndrome.

### CTA protocol

All studies were performed with a 64-row 128-slice CT system (GE Optima CT 660, GE Healthcare, USA) using the following parameters: collimation 39.38 × 0.625 mm, spiral pitch 0.516:1, anode voltage 120 kV, anode amperage 40–500 mA, rotation time 0.6 seconds, slice thickness 0.625 mm. Patients received intravenous non-ionic contrast agent (Iomeron 350, Bracco Imaging Deutschland GmbH, Konstanz, Germany) at volume adjusted to their body weight, 50 mL on average, followed by a 30 mL NaCl flush. Iomeron 350 was given to the basilic vein at a rate of 4–4.5 mL/second with the aid of an automatic syringe (OptiVantage DH, Mallincrodt, St Louis, MO, USA). The scanning started 15–20 seconds after administration of the contrast agent, after reaching attenuation of 100 HU above the baseline attenuation and lasted for 6 seconds. The images were recorded as digital imaging and communications in medicine (DICOM) files on a HP Z800 workstation.

### Morphometric analysis of MCA bifurcations

**Figure 1:**
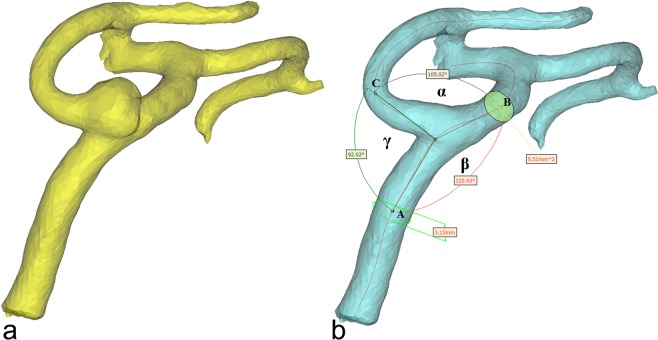
Three-dimensional model of the MCA before (**a**) and after (**b**) a digital removal of the aneurysm, obtained in Mimics v.17.0 based on DICOM files from CTA. The centerline (red line) was automatically fitted to the model. Points A, B and C correspond to the largest curvatures of the main trunk of the MCA and post-bifurcation branches (the larger and smaller branch, respectively); these are the points for which vessel cross-sectional areas and the best-fit diameters were calculated automatically. The arms of α angle were formed by points B and C, and the point at the intersection of both centerlines. β angle, between the MCA trunk and the larger branch, was defined by points A and B, and the point at the intersection of both centerlines. γ angle, between the MCA trunk and the smaller branch, was defined by points A and C, and the point at the intersection of both centerlines.

CTA scans data in DICOM format were transferred to Mimics Innovation Suite (MIS) platform (Materialise, Leuven, Belgium). Image segmentation and creation of three-dimensional (3D) models were carried out with Mimics v.17.0 MIS software (Materialise, Leuven, Belgium). The process of segmentation involved main trunks of the MCA, and the post-bifurcation branches. Trifurcations of the main MCA trunk were excluded from the morphometric analysis. MCA bifurcations from the aneurysm patients were divided into two groups: the An group with 102 aneurysmal MCA bifurcations (96 bifurcations with MCA aneurysm and 9 contralateral bifurcations with mirror aneurysm, excluding 3 aneurysmal trifurcations) and the non-An group with 82 contralateral non-aneurysmal MCA bifurcations (9 bifurcations with mirror aneurysm and 5 MCA trifurcations were excluded from the analysis). Also, MCA bifurcations from the controls were divided into two groups: R-MCA group with 88 bifurcations of the right MCA (6 trifurcations were not included) and the L-MCA group with 87 bifurcations of the left MCA (without 7 trifurcations). Before the morphometric calculations, the MCA aneurysms in the An group were digitally erased using the Mimics software. After 3D model creation, the vessel centerline was fitted automatically with a computer-aided design (CAD) tool (Fig. 1). The other parameters: tortuosity, the largest curvature of MCA main trunk and the largest curvatures of the two post-bifurcations branches were calculated automatically according to the centerline. The points of the largest curvature were set as close to the bifurcation as possible, but in a distance longer than 5 mm. Based on these points, the cross-sectional area and the best fit diameter of the MCA trunk (p_0_ and d_0_, respectively) and its two post-bifurcation branches (p_1_, d_1_ and p_2_, d_2_ for the larger and smaller branch, respectively) were estimated automatically. The best fit diameters were then used to calculate the radii of the main MCA trunk (r_0_) and its branches (r_1_ and r_2_ for the larger and smaller branch, respectively) using the following formula:

1 $${\rm{r}}={\rm{d}}/2$$

where r – radius and d – the best fit diameter. The radii were used to calculate two ratios describing the bifurcation asymmetry:

2 $${\rm{asymmetry}}\,{\rm{ratio}}={{{\rm{r}}}_{2}}^{2}{{{\rm{r}}}_{1}}^{-2}$$

and

3 $${\rm{area}}\,{\rm{ratio}}=({{{\rm{r}}}_{1}}^{2}+{{{\rm{r}}}_{2}}^{2}){{{\rm{r}}}_{0}}^{-2}$$

The centerlines and the largest curvature points were exported to 3-matic v.9.0 MIS software to determine angles between the bifurcation components. Three points of the largest curvatures (the main MCA trunk and two post-bifurcations branches) together with the point of the intersection of both centerlines passing through the main trunk of MCA and both branches determined the arms and the apex of the three angles. The following angle values were automatically calculated: the angle between the post-bifurcation branches (α angle) and the angles between the MCA trunk and the larger and the smaller branches (β and γ angle, respectively; Fig. 1). Finally, the angles between each post-bifurcation branch and the direction of the MCA trunk were calculated as:

4 $${\Phi }_{1}=180-{\rm{\beta }}$$

5 $${\Phi }_{2}=180-{\rm{\gamma }}$$

### Transcranial color-coded sonography (TCCS) protocol

**Figure 2:**
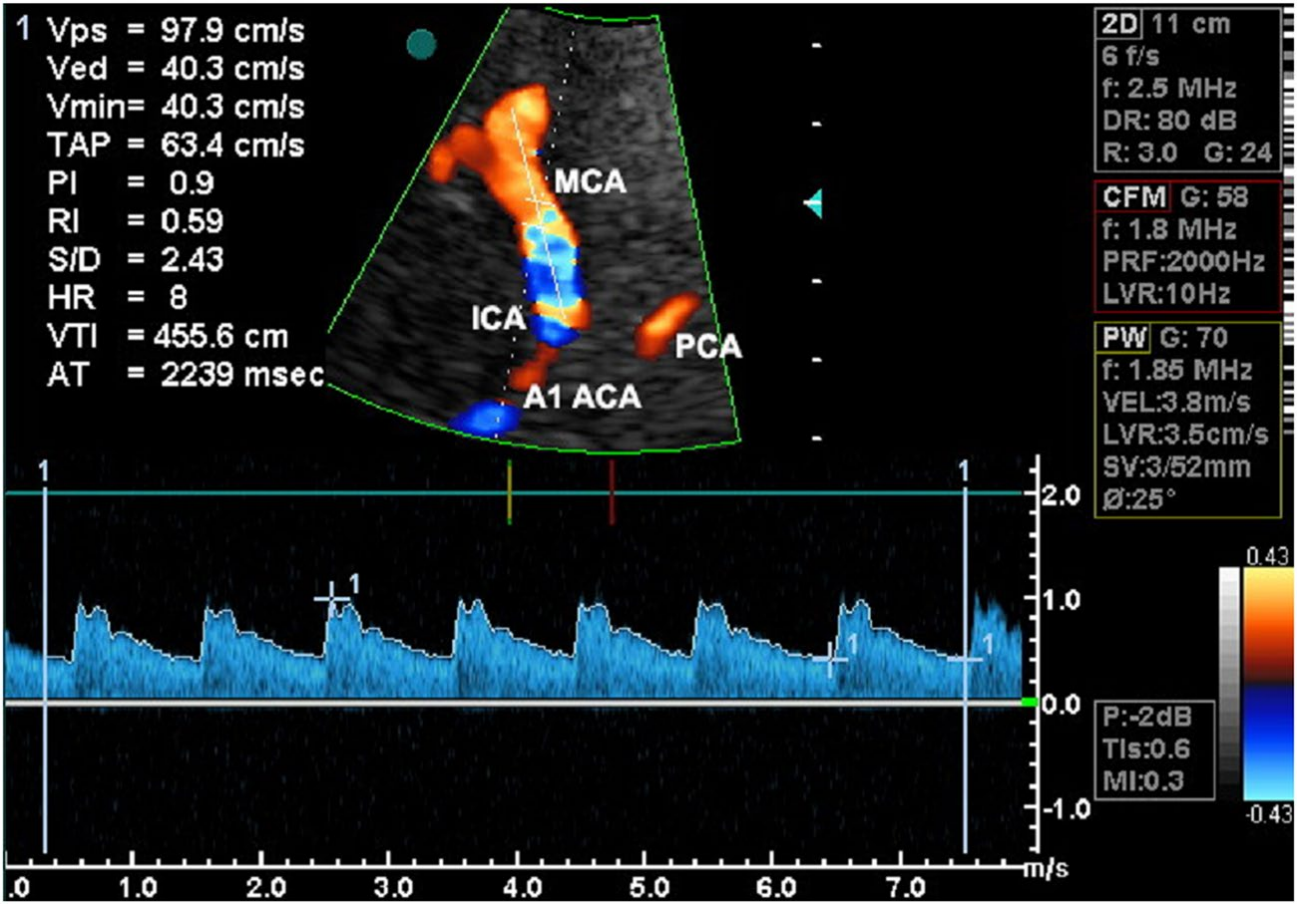
View of a color-coded image of the middle cerebral artery (MCA) with a corresponding Doppler spectral analysis performed using a transtemporal insonation. The sample volume was placed at a depth of 52 mm in the distal portion of the MCA. ICA, the internal carotid artery; PCA, the posterior cerebral artery; A1 ACA, A1 segment of the anterior cerebral artery.

All TCCS examinations were performed by the same researcher using a Vivid 3 Pro (GE Healthcare, Chicago, Illinois, USA) equipped with a multi-frequency transcranial probe (1.5–3.6 MHz), according to the previously described standards^5^. Anterior cerebral circulation was imaged through the temporal acoustic window with the subject in a supine position. Angle-corrected mean blood flow velocity (V_m_), peak systolic velocity (V_ps_) and end-diastolic velocity (V_ed_) were measured for both MCAs (Fig. 2). Pulsatility index (PI) and volume flow rate (VFR) in each vessel were calculated as:

6 $${\rm{PI}}=({{\rm{V}}}_{{\rm{ps}}}-{{\rm{V}}}_{{\rm{ed}}}){{{\rm{V}}}_{{\rm{m}}}}^{-1}$$

7 $${\rm{VFR}}={{\rm{V}}}_{{\rm{m}}}{{\rm{p}}}_{0}$$

where p_0_ – a cross-sectional area of the main MCA trunk.

### Calculation of predicted optimal morphometric parameters

According to the PMW, the adjustment of a given vascular system to its theoretical optimum is expressed as the junction exponent (n):

8 $${{{\rm{r}}}_{0}}^{{\rm{n}}}={{{\rm{r}}}_{1}}^{{\rm{n}}}+{{{\rm{r}}}_{2}}^{{\rm{n}}}$$

where the optimal value of junction exponent for an energetically ideal vessel bifurcation equals 3. The junction exponents for all MCA bifurcations were obtained with an online calculator available at http://www.wolframalpha.com. Optimal angles between the axis of MCA trunk and the larger and smaller post-bifurcation branches (ϕ_1_, ϕ_2_, respectively), as well as the total bifurcation angle (ϕ_1_ + ϕ_2_), were predicted using four PMW-derived optimality rules: minimum surface, minimum volume, minimum pumping power, and minimum endothelial drag rule according to the formulas:

9 $$\cos \,{{\rm{\phi }}}_{1}=({{{\rm{r}}}_{0}}^{2}+{{{\rm{r}}}_{1}}^{2}-{{{\rm{r}}}_{2}}^{2}){(2{{\rm{r}}}_{0}{{\rm{r}}}_{1})}^{-1}$$

10 $$\cos \,{{\rm{\phi }}}_{2}=({{{\rm{r}}}_{0}}^{2}+{{{\rm{r}}}_{2}}^{2}-{{{\rm{r}}}_{1}}^{2}){(2{{\rm{r}}}_{0}{{\rm{r}}}_{2})}^{-1}$$

11 $$\cos ({{\rm{\phi }}}_{1}+{{\rm{\phi }}}_{2})=({{{\rm{r}}}_{0}}^{2}-{{{\rm{r}}}_{1}}^{2}-{{{\rm{r}}}_{2}}^{2}){(2{{\rm{r}}}_{1}{{\rm{r}}}_{2})}^{-1}$$

for minimum surface and minimum endothelial drag rules, and

12 $$\cos \,{{\rm{\phi }}^{\prime} }_{1}=({{{\rm{r}}}_{0}}^{4}+{{{\rm{r}}}_{1}}^{4}-{{{\rm{r}}}_{2}}^{4}){(2{{{\rm{r}}}_{0}}^{2}{{{\rm{r}}}_{1}}^{2})}^{-1}$$

13 $$\cos \,{{\rm{\phi }}^{\prime} }_{2}=({{{\rm{r}}}_{0}}^{4}+{{{\rm{r}}}_{2}}^{4}-{{{\rm{r}}}_{1}}^{4}){(2{{{\rm{r}}}_{0}}^{2}{{{\rm{r}}}_{2}}^{2})}^{-1}$$

14 $$\cos ({{\rm{\phi }}^{\prime} }_{1}+{{\rm{\phi }}^{\prime} }_{2})=({{{\rm{r}}}_{0}}^{4}-{{{\rm{r}}}_{1}}^{4}-{{{\rm{r}}}_{2}}^{4}){(2{{{\rm{r}}}_{1}}^{2}{{{\rm{r}}}_{2}}^{2})}^{-1}$$

for minimum volume and minimum pumping power rules^23^.

### Standard protocol approvals, registrations and patient consent

The protocol of the study was approved by the Institutional Review Board at the Medical University of Silesia in Katowice and all procedures were carried out in accordance with the relevant guidelines and regulations. Written informed consent was sought from all the study participants. The study was registered in the Clinical Trials Registry (ID: NCT03493035).

### Statistical analysis

Normal distribution of the study variables was verified with Shapiro-Wilk test. The results were presented as means and their standard deviations (SD). To assess differences between two independent groups, the Mann–Whitney U test was used. The significance of intragroup differences was verified with Wilcoxon signed-rank test. All morphometric and hemodynamic parameters that showed significant intergroup differences were subjected to logistic regression analysis with stepwise addition mode. The potential predictors of MCA aneurysm were identified on univariate analysis, and the relationship between pairs of the significant predictors was determined based on Pearson’s linear correlation coefficients. The variables with p-values < 0.1 on univariate analysis, except those being correlated with one another, were included in multivariate logistic regression model to identify independent predictors of MCA aneurysm. The results were presented as odds ratios (ORs) and their 95% confidence intervals (CIs). The independent predictors of MCA aneurysm were subjected to receiver operating characteristic (ROC) analysis to identify their cut-off values with optimal sensitivity and specificity. The results were considered statistically significant for p-values < 0.05. Statistical analyses were performed with Statistica v.13.3 package (StatSoft, Tulsa, OK, USA).

## Results

### Morphometric and hemodynamic parameters

Mean values of morphometric and hemodynamic parameters are shown in Table 1. The study groups did not differ significantly in terms of bifurcation asymmetry indices: asymmetry ratio (0.58 vs. 0.62 vs. 0.59 vs. 0.61 for An, non-An, R-MCA and L-MCA groups, respectively) and area ratio (1.09 vs. 1.11 vs. 1.15 vs. 1.14 for An, non-An, R-MCA and L-MCA groups, respectively). Mean r_0_, p_0_, α angle and VFR in An and non-An groups had significantly higher values than in both control groups (r_0_: 1.39 mm and 1.42 mm vs. 1.35 mm and 1.31 mm; p_0_: 6.1 mm^2^ and 6.3 mm^2^ vs. 5.7 mm^2^ and 5.4 mm^2^; α angle: 128.6° and 105.8° vs. 98.6° and 93.1°; VFR: 4.42 cm^3^/s and 4.30 cm^3^/s vs. 3.68 cm^3^/s and 3.46 cm^3^/s for An, non-An, R-MCA and L-MCA groups, respectively). The values of junction exponent and PI for the MCA trunk in An group were significantly lower, and the observed parent-branch angles (Φ_1_, Φ_2_) significantly higher than in both control groups (junction exponent: 2.43 vs. 2.85 and 2.81; PI: 0.81 vs. 0.86 and 0.85; Φ_1_: 58.5° vs. 47.4° and 42.8°; Φ_2_: 82.4° vs. 61.7° and 65.1° for An, R-MCA and L-MCA groups, respectively). No significant differences in these parameters were found between non-An group and both control groups.

**Table 1:** Hemodynamic and Morphometric Parameters for Various Groups of MCA Bifurcations.

Parameter	An (n = 102)	non-An (n = 82)	R-MCA (n = 88)	L-MCA (n = 87)	p-value^a^	p-value^b^	p-value^c^	p-value^d^	p-value^e^
r_0_, mm	1.39 ± 0.18	1.42 ± 0.20	1.35 ± 0.16	1.31 ± 0.19	0.850	<0.05	<0.001	<0.05	<0.001
r_1_, mm	1.16 ± 0.17	1.17 ± 0.20	1.14 ± 0.17	1.11 ± 0.18	0.924	0.645	0.068	0.630	0.080
r_2_, mm	0.84 ± 0.20	0.89 ± 0.17	0.85 ± 0.17	0.84 ± 0.18	0.083	0.998	0.829	0.071	0.052
junction exponent	2.43 ± 0.81	2.86 ± 1.49	2.85 ± 1.13	2.81 ± 1.19	0.268	<0.05	<0.05	0.387	0.642
MCA main trunk tortuosity	0.06 ± 0.04	0.07 ± 0.05	0.07 ± 0.06	0.08 ± 0.07	0.298	0.239	0.295	0.802	0.844
p_0_, (mm^2^)	6.1 ± 1.6	6.3 ± 1.8	5.7 ± 1.2	5.4 ± 1.6	0.863	<0.05	<0.01	<0.05	<0.001
p_1_, (mm^2^)	4.3 ± 1.2	4.3 ± 1.5	4.2 ± 1.2	3.9 ± 1.4	0.770	0.647	0.058	0.928	0.163
p_2_, (mm^2^)	2.3 ± 1.0	2.51 ± 1.1	2.3 ± 0.9	2.3 ± 1.0	0.183	0.926	0.622	0.119	0.065
Asymmetry ratio, r_2_^2^r_1_^−2^	0.58 ± 0.24	0.62 ± 0.22	0.59 ± 0.21	0.61 ± 0.21	0.241	0.674	0.327	0.348	0.733
Area ratio, (r_1_^2^ + r_2_^2^)r_0_^−2^	1.09 ± 0.20	1.11 ± 0.26	1.15 ± 0.22	1.14 ± 0.20	0.688	0.063	0.060	0.292	0.270
Φ_1_ (°)	58.5 ± 24.8	48.3 ± 21.2	47.4 ± 22.9	42.8 ± 22.0	<0.01	<0.01	<0.001	0.869	0.063
Φ_2_ (°)	82.4 ± 20.9	68.1 ± 20.6	61.7 ± 17.1	65.1 ± 18.3	<0.001	<0.001	<0.001	0.064	0.277
α (°)	128.6 ± 24.2	105.8 ± 19.7	98.6 ± 21.4	93.1 ± 18.5	<0.001	<0.001	<0.001	<0.05	<0.001
V_m,_ (cm/s)	70.6 ± 14.1	67.1 ± 14.5	67.0 ± 11.9	66.8 ± 11.5	0.174	0.200	0.139	0.856	0.852
VFR, cm^3^/s	4.42 ± 1.46	4.30 ± 1.54	3.68 ± 0.92	3.46 ± 1.21	0.498	<0.01	<0.001	<0.05	<0.01
PI	0.81 ± 0.11	0.84 ± 0.11	0.86 ± 0.15	0.85 ± 0.14	<0.05	<0.05	<0.05	0.642	0.709

An, aneurysmal MCA bifurcations; non-An, non-aneurysmal contralateral MCA bifurcations; R-MCA, control right-side MCA bifurcations; L-MCA, control left-side MCA bifurcations; r_0_, p_0_, MCA main trunk radius and cross-sectional area; r_1_, p_1_, larger branch radius and cross-sectional area; r_2_, p_2_, smaller branch radius and cross-sectional area; Φ_1_, Φ_2_, α, observed angles between the MCA trunk direction and the larger and the smaller branch and the total bifurcation angle, respectively; V_m_, MCA mean flow velocity VFR, volume flow rate; PI, pulsatility index; ^a^An vs. non-An; ^b^An vs. R-MCA; ^c^An vs. L-MCA; ^d^non-An vs. R-MCA; ^e^non-An vs. L-MCA.

Mean values of Φ_1_, Φ_2_ and α angles in all groups and their optimum values calculated based on the minimum surface rule and minimum endothelial drag rule (ϕ_1_, ϕ_2_, ϕ_1_ + ϕ_2_), or the minimum volume rule and minimum pumping power rule (ϕ′_1_, ϕ′_2_, ϕ′_1_ + ϕ′_2_) are presented in Table 2. The Φ_1_, Φ_2_, α angles in all groups were significantly larger than the predicted optimal angles derived from PMW; the only exception pertained to the observed values of Φ_1_ in L-MCA group and α angle in both control groups, which did not differ significantly from the predicted values calculated based on the minimum surface rule and minimum endothelial drag rule. The difference between predicted and observed bifurcation angles in An group was significantly greater than in non-An group and both control groups (Table 3).

**Table 2:** Predicted Optimal and Observed Bifurcation Angles for Various Groups of MCA Bifurcations.

Group	ϕ_1_ (°)	ϕ′_1_ (°)	Φ_1_ (°)	p-value^a^	p-value^b^	ϕ_2_ (°)	ϕ′_2_ (°)	Φ_2_ (°)	p-value^a^	p-value^b^	ϕ_1_ + ϕ_2_ (°)	ϕ′_1_ + ϕ′_2_ (°)	α (°)	p-value^a^	p-value^b^
An	36.9 ± 9.1	22.4 ±± 10.7	58.5 ± 24.8	<0.001	<0.001	55.8 ± 9.6	39.3 ± 16.6	82.4 ± 20.9	<0.001	<0.001	92.7 ± 12.3	61.7 ± 23.7	128.6 ± 24.2	<0.001	<0.001
non-An	39.0 ± 9.0	24.7 ± 10.9	48.3 ± 21.2	<0.001	<0.001	55.0 ± 10.8	44.8 ± 21.2	68.1 ± 20.6	<0.001	<0.001	94.0 ± 15.4	69.4 ± 26.8	105.8 ± 19.7	<0.001	<0.001
R-MCA	38.5 ± 7.4	23.8 ± 9.3	47.4 ± 22.9	<0.01	<0.001	57.4 ± 9.0	47.8 ± 16.6	61.7 ± 17.1	<0.05	<0.001	95.9 ± 10.5	71.6 ± 20.6	98.6 ± 21.4	0.739	<0.001
L-MCA	38.8 ± 7.7	23.4 ± 10.0	42.8 ± 22.0	0.576	<0.001	56.1 ± 10.0	45.1 ± 21.9	65.1 ± 18.3	<0.001	<0.001	94.9 ± 13.3	68.5 ± 26.8	93.1 ± 18.5	0.095	<0.001

ϕ_1_, ϕ_2_, ϕ_1_ + ϕ_2_; predicted optimal angles calculated based on the rule of minimum surface and rule of minimum endothelial drag; ϕ′_1_, ϕ′_2_, ϕ′_1_ + ϕ′_2_, predicted optimal angles calculated based on the rule of minimum volume and rule of minimum pumping power. For other legends, see Table 1. ^a^ϕ_1_, ϕ_2_, ϕ_1_ + ϕ_2_ and ^b^ϕ′_1_, ϕ′_2_, ϕ′_1_ + ϕ′_2_ vs. Φ_1_, Φ_2_, α, respectively.

**Table 3:** Differences Between Predicted Optimal and Observed Bifurcation Angles for Various Groups of MCA Bifurcations.

Angle	An (n = 102)	non-An (n = 82)	R-MCA (n = 88)	L-MCA (n = 87)	p-value^a^	p-value^b^	p-value^c^	p-value^d^	p-value^e^
ϕ_1_ − Φ_1_ (°)	−21.5 ± 25.0	−8.7 ± 19.6	−8.9 ± 22.9	−3.7 ± 22.6	<0.001	<0.001	<0.001	0.900	0.055
ϕ_2_ − Φ_2_ (°)	−26.6 ± 22.1	−13.7 ± 20.7	−4.0 ± 17.4	−9.1 ± 18.0	<0.001	<0.001	<0.001	<0.01	0.150
(ϕ_1_ + ϕ_2_)-α (°)	−35.9 ± 24.9	−11.8 ± 22.5	−2.4 ± 21.2	2.0 ± 19.9	<0.001	<0.001	<0.001	<0.01	<0.001
ϕ′_1_ − Φ′_1_ (°)	−37.7 ± 22.8	−24.6 ± 21.2	−24.3 ± 23.3	−19.4 ± 25.1	<0.01	0.001	<0.001	0.917	0.121
ϕ′_2_ − Φ′_2_ (°)	−43.8 ± 27.8	−23.8 ± 23.3	−14.6 ± 21.4	−21.5 ± 25.4	<0.001	<0.001	<0.001	0.054	0.701
(ϕ′_1_ + ϕ′_2_)-α (°)	−69.7 ± 29.3	−37.5 ± 31.6	−29.3 ± 25.8	−27.1 ± 32.6	<0.001	<0.001	<0.001	0.091	0.054

For legends, see Tables 1 and 2. ^a^An vs. non-An; ^b^An vs. R-MCA; ^c^An vs. L-MCA; ^d^non-An vs. R-MCA; ^e^non-An vs. L-MCA.

### Predictors of MCA aneurysm: univariate and multivariate analysis

Univariate logistic regression analysis identified r_0_, p_0_, junction exponent, area ratio, Φ_1_, Φ_2_ and α angles, VFR and PI as significant predictors of MCA aneurysm (Table 4). Since r_0_, p_0_ and VFR, area ratio and junction exponent, Φ_1_, Φ_2_ and α angles, V_m_ and VFR correlated with one another, the final multivariate logistic regression model included only junction exponent, α angle, V_m_, PI and VFR. Among these variables, the best predictors of MCA aneurysm were junction exponent (OR = 0.42, 95% CI: 0.24–0.73, p = 0.002), α angle (OR = 1.07, 95% CI: 1.05–1.09, p < 0.001) and VFR (OR = 2.36, 95% CI: 1.47–3.79, p < 0.001) (Table 4).

**Table 4:** Predictors of MCA Aneurysm: Univariate and Multivariate Logistic Regression Analysis.

Variable	Univariate analysis		Multivariate analysis	
	OR (95% CI)	p-value	OR (95% CI)	p-value
r_0_, mm	8.33 (2.00–14.68)	0.004	—	—
p_0_, (mm^2^)	1.30 (1.09–1.54)	0.003	—	—
junction exponent	0.67 (0.51–0.88)	0.004	0.42 (0.24–0.73)	0.002
Asymmetry ratio, r_2_^2^r_1_^−2^	0.62 (0.21–1.89)	0.403	—	—
Area ratio, (r_1_^2^ + r_2_^2^)r_0_^−2^	0.27 (0.08–0.94)	0.039	—	—
Φ_1_ (°)	1.02 (1.01–1.04)	<0.001	—	—
Φ_2_ (°)	1.06 (1.04–1.07)	<0.001	—	—
α (°)	1.06 (1.05–1.08)	<0.001	1.07 (1.05–1.09)	<0.001
V_m_, (cm/s)	1.02 (1.00–1.05)	0.052	—	—
VFR, cm^3^/s	1.65 (1.28–2.11)	<0.001	2.36 (1.47–3.79)	<0.001
PI	0.07 (0.01–0.76)	0.028	—	—

For legends, see Table 1. Multivariate model included all variables with p-value < 0.1 on univariate analysis, except those that were shown to correlate with one another.

### Predictors of MCA aneurysm: ROC analysis

**Figure 3:**
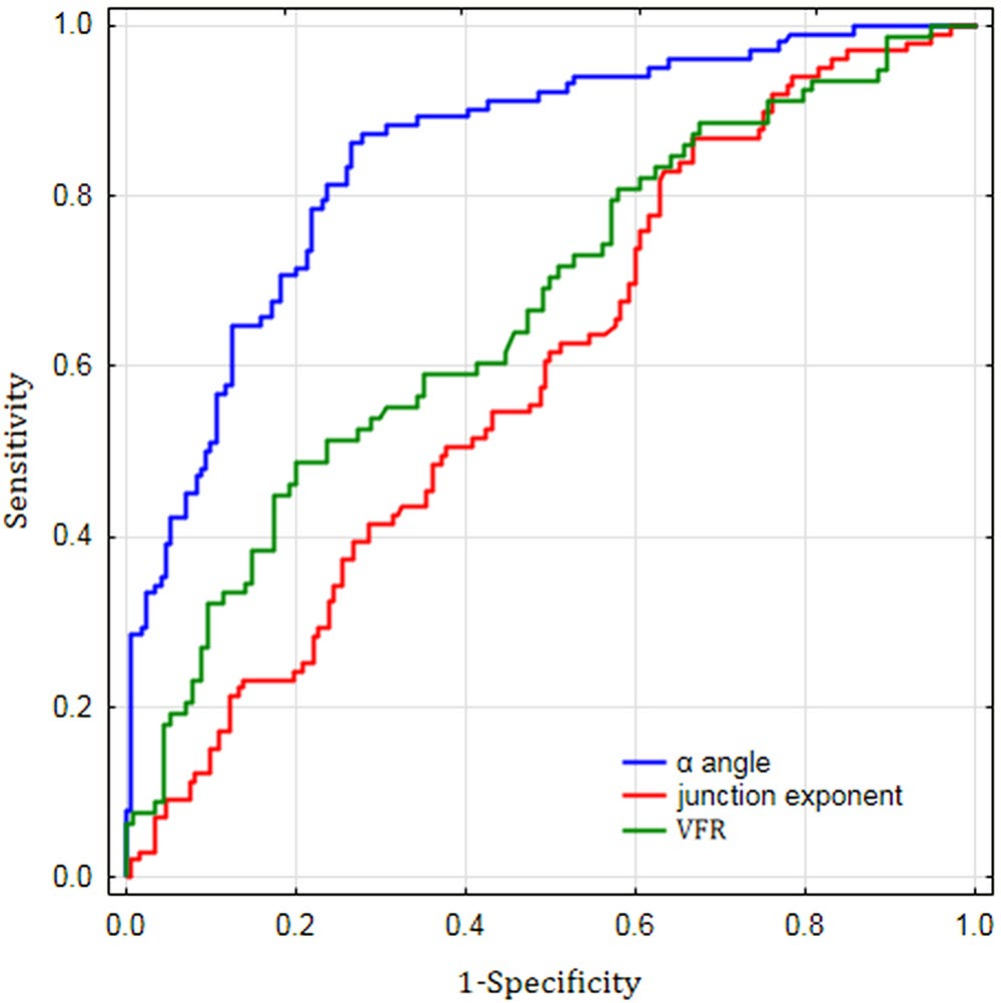
Receiver operator characteristic (ROC) curves for all most significant predictors of MCA aneurysm (description in the text). α angle, the total bifurcation angle; VFR, volume flow rate.

The ROC curve for all independent predictors for MCA aneurysm is presented in Fig. 3. The largest area under curve (AUC) value was observed for α angle (AUC = 0.848), followed by VFR (AUC = 0.667) and junction exponent (AUC = 0.593), which implies that α angle was the most accurate predictor of MCA aneurysm among all variables included in the logistic regression model. The optimal cut-off values for α angle, VFR and junction exponent which most accurately distinguished between aneurysmal and non-aneurysmal bifurcations were 104.5° (sensitivity 0.86, specificity of 0.73), 4.38 cm^3^/s (sensitivity 0.49, specificity of 0.80) and 3.05 (sensitivity 0.87, specificity of 0.33), respectively.

## Discussion

Despite a progress in the research on cerebral aneurysm pathogenesis, the etiology of these malformations is still a matter of debate. With no doubt, hemodynamic stress at arterial bifurcation plays a key role as a trigger of aneurysm formation. In studies conducted by Meng *et al*.^2,24^, involving CFD and histological analysis of canine and rabbit models, a combination of high wall shear stress (WSS) and high WSS gradient at arterial bifurcation was shown to lead to cerebral aneurysm formation by disruption of the internal elastic lamina, loss of smooth muscle cells (SMCs) and a decrease in SMC proliferation rate. Recent evidence suggests than aneurysm formation might be also associated with the influence of multiple genetic factors which determine the strength of the arterial wall, which is further modified by environmental factors, such as smoking and hypertension^25,26^. However, the majority of cerebral aneurysms are located solely in a few arterial bifurcations, i.e. ACoA complex, ICA, MCA and BA, which implies that aside from genetic, hemodynamic and environmental factors, their formation might be also associated with the geometry of the circle of Willis arteries.

### Vascular dimensions of MCA bifurcation

While according to published anatomical data, mean diameter of the MCA trunk is 3.9 mm (range 2.4–4.6 mm)^27^, the value calculated for the same vessel using radiological methods, such as 3D Magnetic Resonance Angiography (MRA), equals 2.2 mm^28^. This discrepancy results from the fact that during anatomical measurements, the outer diameter of the vessel is determined, whereas the inner diameter is calculated during the imaging studies (e.g. MRA, CTA). Although our findings regarding the MCA diameter are consistent with the data presented above, our study groups differed in terms of the MCA trunk diameters. Mean diameters of aneurysmal MCA trunks and contralateral non-aneurysmal MCA trunks were significantly larger than the respective diameters in healthy controls. A relationship between the diameter of main arterial trunk and the risk of aneurysm formation is not straightforward. It is generally believed that enhanced blood flow in arteries with larger radiuses contributes to an increase in hemodynamic stress at the vessel apex, which might eventually contribute to aneurysm development^1,6–8^. However, according to some authors, this is smaller diameter of parent vessel which is associated with higher jet flow at the bifurcation apex and resultant greater hemodynamic stress^29,30^. In our opinion, the role of vessel diameter cannot be discussed separate from hemodynamic parameters, such as blood flow velocity and VFR. The latter two are known to correlate positively with shear stress, a factor responsible for endothelial damage and initiation of aneurysm formation. In our present study, high VFR turned out to be an independent risk factor for aneurysm formation. Importantly, VFR in contralateral MCA trunks of aneurysm patients was also significantly higher than in the control vessels, which might explain the phenomenon of mirror aneurysm development.

Aside from the vessel dimensions, also bifurcation symmetry seems to play an important role in cerebral aneurysm pathogenesis^28,31,32^. The results of most previous studies imply that the greater the bifurcation asymmetry, the higher the risk of aneurysm development. However, in our opinion, the role of bifurcation symmetry in aneurysm formation should be analyzed in conjunction with the PMW assumptions. According mathematical formulas describing the Murray’s law and the formulas for shear stress, quantitative relation between junction exponent and relative shear stress in the branches depends on a branch to parent vessel caliber ratio^33^. Specifically, the shear stress increases in the branches as the junction exponent decreases, as well as when the caliber ratio decreases. In other words, the greater the degree of asymmetry between branch and parent vessel, the greater the increase in shear stress as the value of junction exponent decreases. If the radiuses of bifurcation vessels follow the Murray’s formula with n = 3, both energy expenditure for circulation maintenance and shear stress are the lowest, regardless of the bifurcation asymmetry. Under such conditions, junction exponent should be considered as a measure of bifurcation adjustment to the energetic minimum. Thus, the importance of bifurcation asymmetry as a determinant of shear stress takes on significance if the radiuses of bifurcation vessels do not follow the Murray’s formula (n≠3). In line with these theoretical assumptions, we demonstrated that despite the lack of significant between-group differences in bifurcation symmetry indices, aneurysm patients presented with significantly lower values of junction exponent than the controls. Furthermore, junction exponent turned out to be an independent predictor of aneurysm formation.

Our findings are consistent with the results published by Rossitti^33^, according to whom patients with cerebral aneurysms presented with significantly lower junction exponent values and higher relative shear stress at the post-bifurcation branches than the non-aneurysmal controls. Furthermore, we demonstrated that junction exponent values for control MCA bifurcations were close to the theoretical optimum of 3 (2.85 and 2.81 for the right and left MCA bifurcations, respectively). Interestingly, the same referred also to the junction exponent values for contralateral MCA bifurcations (non-An group), amounting to 2.86. This may explain why the contralateral non-aneurysmal MCA bifurcations were free from aneurysm although the radiuses of the main MCA trunks and VFR values in non-An group were significantly higher than in the controls.

### MCA bifurcation angles

Most previous imaging-based studies (e.g. 3D rotational angiography, MRA, CTA) dealing with the problem of bifurcation angle morphology showed that wide bifurcation angle constitutes a significant risk factor for aneurysm formation. Those studies included the bifurcations which are generally considered to be predisposed for aneurysm development: ACoA complex^8,16,34–36^, BA^14,29,37^ and MCA^30,38^. Our findings are consistent with the results of the studies mentioned above; our present study showed that Φ_1_, Φ_2_ and α angles in aneurysmal MCA bifurcations were significantly wider than in contralateral and control bifurcations. Additionally, α angle turned out to be an independent predictor of MCA aneurysm, with the cut-off value optimally distinguishing between aneurysmal and non-aneurysmal bifurcations equal 104.5°. These findings are in pair with the results published by Baharoglu *et al*.^38^ according to whom, wide total bifurcation angle (Φ_1_ + Φ_2_) = 140° was the best performer in discriminating between aneurysmal and non-aneurysmal MCA bifurcations (93% sensitivity and specificity).

Importantly, non-aneurysmal MCA bifurcations analyzed in our present study had significantly higher values of α angle than the control bifurcations. These results correspond to those reported by other authors, according to whom patients with aneurysms presented with significantly wider non-aneurysmal bifurcation angles than the healthy controls^14,38^. This implies that persons with aneurysms might also show significant alterations in vascular dimensions and bifurcation angles of other arteries, and thus, might be predisposed to formation of *de novo* or multiple aneurysms. A key to the link between wider bifurcation angle and higher risk of cerebral aneurysm formation might be found in the results of CFD studies. In those studies, wider bifurcation angle was associated with abnormally enhanced hemodynamic stresses, enlarged zones of direct flow impingement and larger peak pressure area; this contributed to vessel wall damage at the apex and eventually lead to aneurysm formation^13,16^.

### Summary

According to PMW, optimal dimensions and bifurcation angles of cerebral vessels are essential to preserve minimum expenditure along the network by maintaining constant shear stress across daughter and parent vessels. Therefore, each local change in vascular geometry results in greater shear stress at the bifurcations, which propagates through all the ramifications of the network. This, in turn, might predispose to various pathologies including cerebral aneurysms^33^. Our findings seem to support these theoretical assumptions, as we demonstrated that the dimensions of the bifurcation vessels in patients with cerebral aneurysms, but not in the non-aneurysmal controls, did not follow the PMW. Moreover, we showed that the MCA bifurcation angle in patients diagnosed with cerebral aneurysm was significantly larger than in the controls. Such a result is sufficient to conclude that aside from the deviations in vessel dimensions, also a large bifurcation angle might be an additional risk factor for cerebral aneurysm development. Furthermore, we showed that observed value of the aneurysmal bifurcation angle was significantly larger than the predicted optimal angle derived from the PMW, and the difference between the two was significantly higher in patients with aneurysm than in the controls. These findings imply that the risk of aneurysm development increases with the difference between the observed and predicted optimal value of the bifurcation angle.

Although many angiographic studies demonstrated that vessel dimensions are governed by the PMW^19–22,32,33,39^, several of them showed that the bifurcation angles do not follow this rule^19,21,22,40^. In our present study, observed values of the bifurcation angle turned out to be significantly higher than the predicted optimal values in all study groups, including the controls. This means that unlike for the arterial dimensions, the geometry of arterial bifurcations, whether in aneurysm patients or in non-aneurysmal controls, deviated from a PMW-derived optimum. Hence, in line with the PMW provisions, the deviations from the theoretical optimal values of the bifurcation angles likely contributed to a greater hemodynamic stress at arterial bifurcations in both patients and controls, predisposing both groups to aneurysm development. On the other hands, the results of some theoretical analyses suggest that even considerable deviations from the optimal angles may result in a relatively low (2–5%) increase in energy cost^40^. Given those discrepancies, further extensive large-scale CFD studies involving true models of the circle of Willis’ vessels are needed to better understand and explain the exact relationship between the bifurcation angle and the magnitude of WSS at the bifurcation apex.

### Limitations

A few limitations of present study need to be addressed. First, we examined patients who had already been diagnosed with the MCA aneurysms. Thus, it cannot be excluded that at early stages of the aneurysm formation, morphometric and hemodynamic parameters of the MCA bifurcation might have been slightly different than at the time of the study. Second, although the MCA aneurysms were erased from the vascular images before the morphometric analysis, their presence could interfere with the bifurcation geometry. In our opinion, however, it had little impact on the results, since most patients had small MCA aneurysms, with a mean diameter of 5.0 ± 3.4 mm (range 2.0–26.4 mm). Third, the study might have also suffered from a selection bias. Although the participants were enrolled prospectively, some patients with aneurysms missed on CT scans due to their small size might have been inadvertently excluded.

## Conclusions

This study demonstrated that development of cerebral aneurysms might be an independent effect of abnormalities in hemodynamic and morphometric factors. The most important risk factors for aneurysm development were wide bifurcation angle, high-volume blood flow in the parent vessel and low junction exponent value. The risk of aneurysm increased proportionally to the deviation of morphometric parameters of the bifurcation vessels from their optimal PMW-derived values. The role of bifurcation angle in aneurysm development needs to be explained in future research as the values of this parameter in both aneurysm patients and non-aneurysmal controls in were scattered considerably around the PMW-derived optimum.

## Data Availability

Fully anonymized data not published within this article will be made available by request from any qualified investigator following the EU General Data Protection Regulation.

## References

[1] Stehbens WE (1989). Etiology of intracranial berry aneurysms. J. Neurosurg..

[2] Meng H (2007). Complex hemodynamics at the apex of an arterial bifurcation induces vascular remodeling resembling cerebral aneurysm initiation. Stroke.

[3] Ferguson GG (1972). Physical factors in the initiation, growth, and rupture of human intracranial saccular aneurysms. J. Neurosurg..

[4] Dyste GN, Beck DW (1989). De novo aneurysm formation following carotid ligation: case report and review of the literature. Neurosurgery.

[5] Kaspera W, Majchrzak H, Ladzinski P, Tomalski W (2005). Color doppler sonographic evaluation of collateral circulation in patients with cerebral aneurysms and the occlusion of the brachiocephalic vessels. Neurosurgery.

[6] Stehbens WE (1963). Aneurysms and anatomical variation of cerebral arteries. Arch. Pathol..

[7] Kayembe KN, Sasahara M, Hazama F (1984). Cerebral aneurysms and variations in the circle of Willis. Stroke.

[8] Kaspera W (2014). Morphological, hemodynamic, and clinical independent risk factors for anterior communicating artery aneurysms. Stroke.

[9] Hashimoto N, Handa H, Hazama F (1978). Experimentally induced cerebral aneurysms in rats. Surg. Neurol..

[10] Hashimoto N (1987). Experimental induction of cerebral aneurysms in monkeys. J. Neurosurg..

[11] Roach MR, Scott S, Ferguson GG (1972). The hemodynamic importance of the geometry of bifurcations in the circle of Willis (glass model studies). Stroke.

[12] Ujiie H (1996). Hemodynamic study of the anterior communicating artery. Stroke.

[13] Alnaes MS (2007). Computation of hemodynamics in the circle of Willis. Stroke.

[14] Tütüncü F (2014). Widening of the basilar bifurcation angle: association with presence of intracranial aneurysm, age, and female sex. J. Neurosurg..

[15] Sasaki Tetsuo, Kakizawa Yukinari, Yoshino Masato, Fujii Yasuhiro, Yoroi Ikumi, Ichikawa Yozo, Horiuchi Tetsuyoshi, Hongo Kazuhiro (2018). Numerical Analysis of Bifurcation Angles and Branch Patterns in Intracranial Aneurysm Formation. Neurosurgery.

[16] Zhang XJ (2018). Enlarged anterior cerebral artery bifurcation angles may induce abnormally enhanced hemodynamic stresses to initiate aneurysms. World Neurosurg..

[17] Murray CD (1926). The physiological principle of minimum work applied to the angle of branching of arteries. J. Gen. Physiol..

[18] Murray CD (1926). The physiological principle of minimum work: I. the vascular system and the cost of blood volume. Proc. Natl. Acad. Sci. USA.

[19] Hutchins GM, Miner MM, Boitnott JK (1976). Vessel caliber and branch-angle of human coronary artery branch-points. Circ. Res..

[20] Zamir M, Chee H (1986). Branching characteristics of human coronary arteries. Can. J. Physiol. Pharmacol..

[21] Rossitti S, Löfgren J (1993). Vascular dimensions of the cerebral arteries follow the principle of minimum work. Stroke.

[22] Ingebrigtsen T (2004). Bifurcation geometry and the presence of cerebral artery aneurysms. J. Neurosurg..

[23] Zamir M (1976). Optimality principles in arterial branching. J. Theor. Biol..

[24] Metaxa E (2010). Characterization of critical hemodynamics contributing to aneurysmal remodeling at the basilar terminus in a rabbit model. Stroke.

[25] Zhou S (2018). Genome-wide association analysis identifies new candidate risk loci for familial intracranial aneurysm in the French-Canadian population. Sci. Rep..

[26] Zhou S, Dion PA, Rouleau GA (2018). Genetics of intracranial aneurysms. Stroke.

[27] Gibo H, Carver CC, Rhoton AL, Lenkey C, Mitchell RJ (1981). Microsurgical anatomy of the middle cerebral artery. J. Neurosurg..

[28] Sadatomo T (2013). Differences between middle cerebral artery bifurcations with normal anatomy and those with aneurysms. Neurosurg. Rev..

[29] Can A, Mouminah A, Ho AL, Du R (2015). Effect of vascular anatomy on the formation of basilar tip aneurysms. Neurosurgery.

[30] Can A, Ho AL, Dammers R, Dirven CM, Du R (2015). Morphological parameters associated with middle cerebral artery aneurysms. Neurosurgery.

[31] Bor AS, Velthuis BK, Majoie CB, Rinkel GJ (2008). Configuration of intracranial arteries and development of aneurysms: A follow-up study. Neurology.

[32] Baharoglu MI, Lauric A, Wu C, Hippelheuser J, Malek AM (2014). Deviation from optimal vascular caliber control at middle cerebral artery bifurcations harboring aneurysms. J. Biomech..

[33] Rossitti S (1998). Shear stress in cerebral arteries carrying saccular aneurysms. Acta Radiol..

[34] Kasuya H (1999). Angles between a1 and a2 segments of the anterior cerebral artery visualized by three-dimensional computed tomographic angiography and association of anterior communicating artery aneurysms. Neurosurgery.

[35] Ye J, Zheng P, Hassan M, Jiang S, Zheng J (2017). Relationship of the angle between the A1 and A2 segments of the anterior cerebral artery with formation and rupture of anterior communicating artery aneurysm. J. Neurol. Sci..

[36] Zhang XJ, Gao BL, Hao WL, Wu SS, Zhang DH (2018). Presence of anterior communicating artery aneurysm is associated with age, bifurcation angle, and vessel diameter. Stroke.

[37] Zhang XJ (2018). Association of basilar bifurcation aneurysms with age, sex, and bifurcation geometry. Stroke.

[38] Baharoglu MI (2014). Widening and high inclination of the middle cerebral artery bifurcation are associated with presence of aneurysms. Stroke.

[39] Rossitti S (1995). Energetic and spatial constraints of arterial networks. Arq. Neuropsiquiatr..

[40] Zamir M, Bigelow DC (1984). Cost of departure from optimality in arterial branching. J. Theor. Biol..

